# A school-based intervention program in promoting leisure-time physical activity: trial protocol

**DOI:** 10.1186/s12889-018-5320-1

**Published:** 2018-04-02

**Authors:** Masato Kawabata, Khai Leng Chua, Nikos L. D. Chatzisarantis

**Affiliations:** 1 Physical Education and Sports Science Academic Group, National Institute of Education, Nanyang Technological University, 1 Nanyang Walk, Singapore, 637616 Singapore; 2 School of Human Movement and Nutrition Sciences, The University of Queensland, Brisbane, QLD 4072 Australia; 3 School of Psychology and Speech Pathology, Faculty of Health Sciences, Curtin University of Technology, Perth, WA 6102 Australia

**Keywords:** Moderate-to-vigorous physical activity, Prioritization, Leisure-time, Physical education, Theory of planned behavior

## Abstract

**Background:**

Regular participation in moderate-to-vigorous physical activity (MVPA) is important to manage obesity. Physical education (PE) is considered to play an important role in promoting lifelong participation in physical activity (PA) because it provides an existing network where cost-effective interventions can be implemented to produce sustainable change in health behavior. However, the association between compulsory school PA (e.g., PE lessons) and body composition levels has received mixed support in the literature. Therefore, the present study aimed to investigate whether a school-based intervention targeting salient PA benefits and barriers grounded on the theory of planned behavior would promote young people’s participation in MVPA during leisure time and reduce body mass index (BMI) of overweight students.

**Methods/design:**

A total of 171 students from 3 secondary schools in Singapore underwent the control condition followed by the intervention condition. Both the conditions consisted of PE lessons twice per week over 4 weeks. In the control condition, PE teachers encouraged students to participate in PA during leisure time without providing persuasive message. While in the intervention condition, PE teachers delivered persuasive messages that targeted the salient benefits and barriers associated with PA to the students at the last 5 to 10 min of each PE lesson. PA levels over a week were measured objectively with wrist-mounted GENEActiv Original accelerometers and subjectively with self-reporting questionnaires three times (Baseline, Post 1, and Post 2) in each condition. Student’s self-reported PA level was measured using the Leisure-Time Physical Activity Participation Questionnaire and the International Physical Activity Questionnaire, and their attitudes, intentions, subjective norms and perceived behavior control towards leisure-time PA were measured with a questionnaire based on the theory of planned behavior. Furthermore, students’ intention, determination and willingness to engage in leisure-time PA were compared with the other activity (e.g., doing homework, shopping).

**Discussion:**

This study will provide the evidence on the effectiveness of a cost-effective school-based intervention on reducing BMI of overweight students through promoting sustained participation in leisure-time PA. It will also address methodological issues on the gaps between objective and subjective measures of PA.

**Trial registration:**

This trial is registered with the ISRCTN registry (ISRCTN73786157, 26/10/2017, retrospectively registered).

## Background

Ministries and organizations worldwide have recognized that adopting moderate-to-vigorous physical activity (MVPA) is an important strategy to help manage obesity while at the same time improves mood by assisting individuals to cope with stress [1]. There is a close relationship between physical activity (PA) and productivity where individuals who engaged in regular PA experience higher levels of psychological well-being and are more productive in school or at workplace as compared to individuals who are physically inactive [2].

Physical education (PE) in schools is considered to play an important role in promoting lifelong participation in PA because it provides an existing network where cost-effective interventions can be implemented to produce sustainable change in health behavior [3–5]. In fact, one of PE goals highlighted by Singapore Ministry of Education is associated to the benefits of living a physically active and healthy life through regular participation in PA [6]. However, teachers and health professionals have found it challenging to motivate children to engage in PA via school-based interventions [7, 8]. A study [9] demonstrated that provision of compulsory school PA was not associated with participation in PA, fitness or body composition levels (e.g., body mass index [BMI]) in adulthood. Findings of systematic review and meta-analytic studies also rose questions about the value of school programs on improving BMI [10, 11].

### Theoretical framework

Interventions are likely to be successful with motivating individuals to participate in PA when they are based on rigorous theory and their content is informed by theories of human motivation [12, 13]. This is supported by a growing body of literature that shows that theory-based interventions produce more sustainable changes in leisure-time PA when compared to interventions that are not based on theory [14]. For example, intervention programs that were based on the theory of planned behavior (TPB) were found to be effective in promoting PA [15–18]. Although intervention programs based on the TPB have been conducted to university students, adults or the senior citizens, there is relative dearth of research that targeted the promotion of health behaviors in classroom settings. To fill the gaps in literature, it is useful to develop a school-based intervention program by adopting Ajzen’s TPB [19] and evaluate its effectiveness in promoting MVPA during leisure time.

Only a few studies have been conducted to test the effectiveness of the TPB in promoting PA during leisure time among young people [20, 21]. Consistent with the theory, the studies showed that the effectiveness of interventions depends on two factors: (a) information saliency and (b) the types of beliefs communicated during intervention periods. As for the first factor, school-based interventions were found to be effective when they communicated salient information that adolescents “felt” personally relevant or important to themselves [20, 21]. For example, persuasive messages that emphasized on enjoyment of leisure-time PA elicited more positive responses than those that emphasized on reducing the risk of cardiovascular diseases since adolescents perceived getting a heart attack as a remote prospect for them [20].

**Fig. 1:**
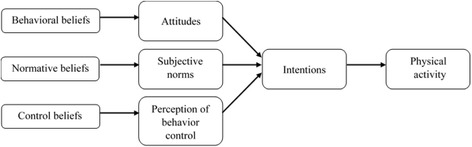
Model of the theory of planned behavior

The second factor is related to the types of beliefs communicated during intervention periods. According to the TPB, interventions may target behavioral beliefs by emphasizing salient benefits or de-emphasize disadvantages associated with PA through attitudes (individuals’ positive vs. negative evaluations toward PA) and intentions (the extent to which individuals are willing to exert effort towards performing PA). Alternatively, interventions may target normative beliefs which emphasize the attitudes of significant others (e.g., attitudes of teachers or parents) towards PA. The interventions that target normative beliefs are likely to influence PA participation through subjective norms (individuals’ beliefs about whether significant others approve or disapprove PA participation) and intentions. Further, interventions may assist people to overcome salient barriers by targeting control beliefs. Interventions that target control beliefs are likely to affect PA participation through perceptions of their behavior control (individuals’ beliefs about whether they possess the necessary resources or skills to perform PA or overcome barriers) and intentions. The model underpinning the TPB is presented in Fig. 1. A pilot study was recently conducted to examine the effectiveness of a school-based program in promoting leisure-time PA for secondary school students [21]. The intervention program targeted on salient benefits and barriers to doing PA. To examine the duration of the intervention program, it was conducted over 8 weeks to half of the participants but only for 4 weeks to the other half of the participants. Results of the pilot study revealed that regardless of the intervention duration, the average leisure-time PA increased from the baseline to the midpoint of the intervention period (4 weeks after the baseline) and it was maintained until the end of the program (8 weeks after the baseline). Although these results were encouraging, the pilot study had several limitations. First, the usefulness and cost-effectiveness of the intervention program on reducing BMI were not evaluated. Second, the PA participation was measured only with self-reported questionnaires and the sustained leisure-time PA level was not assessed with an objective measure. Finally, there was no comparison on the effectiveness of the intervention with those who did not receive an intervention as the pilot study did not include a control group.

### Aims of the study

The present study aimed to investigate whether a school-based intervention targeting salient PA benefits and barriers grounded on the TPB would promote young people’s participation in MVPA during leisure time and reduce BMI of overweight students.

It was hypothesized that a) the proposed school-based intervention program grounded on the TPB would be useful to promote young people’s participation in MVPA during leisure time (*H*_*1*_), b) overweight student’s BMI would be reduced after the intervention, compared to their BMI in the control condition (*H*_*2*_), and c) the effects of the intervention program on PA intentions and participation as well as BMI would be medicated by students’ attitudes and perceptions of behavior control (*H*_*3*_).

## Methods/design

### Study design

**Fig. 2:**
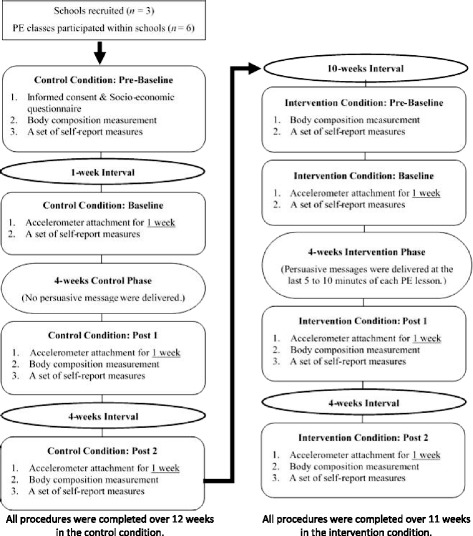
Flow of study design

The Institutional Review Board at Nanyang Technological University (Singapore) provided ethical approval for the current study. Although the study was originally planned to conduct as a cluster-randomized controlled trial, the original plan had to be changed due to great difficulty in recruiting schools. This study adopted a 2 (type of conditions: control vs. intervention targeting salient PA benefits and barriers) × 3 (measurement points: Baseline, Post 1 and Post 2) within-subjects design in which all participants underwent the control condition followed by the intervention condition. Figure 2 provides the flow of the study design.

### Participants

A total of 171 students aged 13 to 16 years old and attending co-educational secondary schools voluntary participated in the present study. All secondary schools in Singapore were invited to participate in the study via e-mail through publicly available contact details. The research team also attended a meeting with school principals to invite their schools to participate in the study. The three secondary schools whose principal granted permission were included in the study, and approval for data collection at the schools was also obtained from Singapore Ministry of Education. After the department head and PE teachers selected two classes at their school, a researcher attended their PE lessons and explained the risks and benefits of the study to the students by distributing information sheets and informed consent forms. They also had the opportunity to ask questions about the study requirements prior to completing and signing an informed consent form. Students were asked to pass the information sheets and informed consent forms to their parents and discuss their participation in the study with them. Students who gave their consent and returned the informed consent forms signed by their parent were eligible to participate in the study. Parents who consented were asked to complete a brief demographics and socioeconomic status questionnaire. Students who did not return the signed informed consent forms or could not participate in PE lessons were excluded from the study. They participated in PE lessons together with their classmates, but did not do any tasks for the study.

The pilot study by Chatzisarantis et al. [21] reported that their TPB-based intervention with mixed (between and within-subjects) design had a small effect on adolescents’ PA participation (ƞ_p_^2^ = .01) and large effects on their beliefs (ƞ_p_^2^ = .15). Therefore, a conservative small effect size of ƞ_p_^2^ = .02 (*f* = .14) was adopted to calculate the total sample size with G*Power version 3.1.9.2 [22]. It was estimated that a sample of 174 participants was required to achieve a power of .90 at the alpha level of .05 for the repeated within-subjects analysis of 6 groups (2 classes at three schools) and 6 measurement points (3 measurement points in two conditions).

### Procedure

Prior to the commencement of the study in each condition, students’ body compositions (e.g., height, body mass and body fat percentage) were measured at their school. Height was measured using a ruler attached to the wall to the nearest 0.5 cm, body mass and body fat percentage were measured with a body composition monitor (Omron Karada Scan: HBF-362). Students were also asked to complete a demographics sheet and a set of self-report measures of leisure-time PA and psychological variables (Pre-Baseline). After completing the questionnaire set, students were instructed to wear the GENEActiv (Original) accelerometer (Activinsights Ltd, Cambridgeshire, UK) on their non-dominant wrist for one week to objectively measure frequency and intensity of their PA level (Baseline). When students returned the accelerometer, they were also asked to complete another set of the same self-report measures (Baseline). Subsequently, students spent four weeks in the control or intervention condition. During the 4 weeks, an intervention program was conducted in PE lessons in the intervention condition. After the 4 weeks, students were requested to wear the accelerometer again for one week across two occasions (Post 1 and Post 2) with a 4 weeks interval period in between before measuring their body compositions and completing the same set of self-report measures. Students’ height, body mass and body fat percentage were measured at three occasions (Pre-Baseline, Post 1 and Post 2) in each condition (see Fig. 2).

### Intervention and fidelity check

PE lessons were conducted twice a week over 4 weeks in both control and intervention conditions. In the control condition, PE teachers encouraged students to participate in PA during leisure time without delivering persuasive messages in PE lessons. An intervention program was implemented after the control period was over (see Fig. 2).

Prior to the intervention period, PE teachers were trained on how to deliver persuasive messages through a 3-h workshop. During the workshop, PE teachers were encouraged to express their concerns about delivering persuasive messages, researchers addressed those concerns to assist PE teachers to conduct the intervention at a sufficient level of proficiency. The persuasive messages targeted salient information related to the benefits and barriers associated with PA, which were identified based on the results of surveys conducted for students at Pre-Baseline in the control condition. Some examples of salient benefits were “health benefits”, “improving physical skills and fitness”, “fun and socialization” and “recommended physical activity level”, whereas salient barriers were “lack of time”, “lack of facilities or equipment”, “feeling tired” and “weather”.

In the intervention condition, PE teachers delivered the persuasive messages that targeted the salient benefits and barriers associated with PA at the last 5 to 10 min of each PE lesson. After PE teachers delivered the persuasive message, students were asked to answer a question about the message in each lesson (e.g., “Please explain briefly how do you know that MVPA improves your fitness levels?”). Researchers evaluated PE teachers’ fidelity of the interventions with a checklist (e.g., “The PE teacher provided clear instructions on this session’s message”) on a scale ranging from 1 (*not at all*) to 5 (*very much*).

### Outcome measures

Students’ BMI were calculated based on their height and body mass, and overweight or obese students were identified based on Cole et al.’s classification [23]. Students’ PA levels were measured objectively with GENEActiv (Original) accelerometers configured at 100 Hz, and subjectively with the International Physical Activity Questionnaire [24] (e.g., “During the last 7 days, how many days did you do vigorous physical activities?”),and the Leisure-Time Physical Activity Participation Questionnaire [25] (“How often did you do any regular activity long enough to sweat last week during your leisure time?”) on a 8-point scale ranging from 0 (*none*) to 7 (*every day*).

Students’ attitudes, intentions, subjective norms and perceived behavior control towards leisure-time PA were measured with a questionnaire based on the constructs from the TPB. Attitudes were assessed through five items with bipolar adjectives (enjoyable/unenjoyable, good/bad, useful/useless, interesting/boring, and beneficial/harmful) on a 7-point semantic differential scale [26]. Behavioral intentions were assessed with three items (e.g., “For the next 4 weeks, I intent to do vigorous physical activity for at least 3 times [*at least 30 minutes*] per week.”) based on a 7-point scale ranging from 1 (*unlikely*) to 7 (*very likely*) [19]. Subjective norms were assessed through three items (e.g., “Most people who are important to me think I should do vigorous physical activity for at least 3 times [*at least 30 minutes*] per week for the next 4 weeks.”) based on a 7-point scales ranging from 1 (*strongly disagree*) to 7 (*strongly agree*) [21]. Perceptions of behavior control were measured using three items (e.g., “I feel in complete control over doing vigorous physical activities for at least 3 times [*at least 30 minutes*] per week for the next 4 weeks.”) based on 7-point scale ranging from 1 (*completely false*) to 7 (*completely true*) [19].

Participants were also asked to compare intention, determination and willingness to engage in another activity during their leisure time with vigorous PA and answer three questions (e.g., “How much do you intend to do the other activity for at least 30 minutes, 3 times a week for the next 4 weeks during your leisure time?”) based on a 7-point scale, ranging from 1 (*not at all*) to 7 (*very much*).

Goal conflict and facilitation were assessed through five items (e.g., “To what extent is the other activity likely to prevent you from doing vigorous physical activity for at least 30 minutes, 3 times a week for the next 4 weeks during your leisure time?”) based on a 7-point scale ranging from 1 (*not at all*) to 7 (*very much*).

### Data analysis

To examine the first research hypothesis (*H*_*1*_), a 2 (type of conditions: control vs. intervention) × 3 (measurement points: Baseline, Post 1 and Post 2) repeated ANOVA will be conducted on students’ MVPA levels. The second hypothesis (*H*_*2*_) will be examined through a 2 (type of conditions: control vs. intervention) × 3 (measurement points: Pre-Baseline, Post 1 and Post 2) repeated ANOVA on students’ BMI levels. A path analysis will be conducted to examine the third hypothesis (*H*_*3*_) with *M*plus (Version 8) [27]. Student’s age, gender, socio-economic status, and prioritization of PA together with fidelity of intervention programs will be included as covariates in the analyses. Furthermore, correlation analyses will be conducted to examine the relationships between the subjective and objective measures of PA.

A cost effectiveness analysis of the program will also be conducted by focusing on the incremental cost-effectiveness of the intervention condition compared to the control condition. Incremental cost-effectiveness ratios will be calculated in terms of the incremental cost per unit BMI reduction. Differences in BMI between different measurement points (Pre-Baseline, Post 1 and Post 2) will also be calculated. Height will be added as a covariate in the analyses in order to account for the growth change in height over the data collection period. Incremental cost-effectiveness ratios will be calculated using standard techniques coupled with bootstrapping methodology and acceptability curves [28]. Through these analyses, it will provide information about the probability that an intervention is cost-effective.

## Discussion

Regular participation in MVPA is well recognized as an important strategy to help manage obesity [1]. PE is considered to play an important role in promoting lifelong PA participation because it provides an existing network where cost-effective interventions can be implemented to produce sustainable change in health behavior [3–6]. However, the relationship between compulsory school PA (e.g., PE lessons) and body composition levels has received mixed support in the literature [9–11]. A growing body of literature shows that theory-based interventions produce more sustainable changes in leisure-time PA when compared to interventions that are not based on theory [14]. Intervention programs based on the TPB were found to be effective in promoting PA [15–18]. However, only a few studies have been conducted to test the effectiveness of the TPB in promoting PA during leisure time among young people [20, 21]. To fill the gaps in literature, this study was conducted to develop cost-effective novel procedures according to the previous TPB-based intervention studies and investigate the effectiveness of a school-based intervention that targeted salient PA benefits and barriers on promoting secondary students’ leisure-time MVPA as well as reducing BMI of overweight students.

The current study will make a unique contribution to literature by addressing the following research issues. First, it will provide a rigorous test of the effects of a PA intervention on BMI because reductions of BMI are more likely to be observed when individuals engage in MVPA over a prolonged period of time. Establishing a link between school-based intervention programs and BMI is an important finding because the association received mixed support in previous studies [10, 11]. The cost-effectiveness analysis in the current project is also novel because previous studies have not compared the effectiveness of TPB-based school interventions against costs [20, 21].

Apart from establishing a link between school-based intervention programs and BMI, the present study will advance scientific knowledge. Ajzen [19] originally stated that the TPB was designed to predict or motivate behaviors and not necessarily behavioral outcomes such as BMI. In this study, however, we consider BMI as a health outcome that is affected by the school-based intervention program. Hence, if the current intervention is found to be effective in reducing BMI (via PA participation), then such a finding would show that the impact of the TPB could be extended to behavioral outcomes such as BMI.

Furthermore, the effectiveness of the school-based intervention program on promoting the participation in MVPA during leisure time will be rigorously examined by using objective PA data measured by the accelerometers as well as by assessing the fidelity of interventions provided by PE teachers. The self-reported measures were used in the previous TPB-based studies with adolescents [20, 21]. As the MVPA level was measured with both objective and subjective measures, the effectiveness of the present TPB-based intervention program on promoting the leisure-time MVPA level will also be compared with the previous TPB-based studies. In doing so, PA measurement issues between objective and subjective instruments can also be addressed in the current study.

### Implications of study findings

The findings of this study would have significant contribution for schools and physical educators to manage obesity through promoting regular MVPA participation. This is important because it will address the challenges faced by teachers and health professionals in motivating children to engage in PA via school-based interventions. If the intervention program is effective in increasing PA participation relative to the control condition, the present research project could have important implications for education because such a finding would indicate that it is possible to maximize effectiveness of existing school programs (e.g., PE lessons) through brief intervention sessions that can be easily incorporated in lessons. This is a significant implication, considering that school programs are not always effective in promoting sustained leisure-time PA participation [9]. Finally, the cost-effectiveness analysis will also provide important information to policy-makers about whether the current intervention program is valuable economically.

## References

[1] Australian National Preventative Health Agency: Report to the Australian government minister for health*.* Canberra; 2013.

[2] Cadilhac D, Cumming T, Sheppard L, Pearce D, Carter R, Magnus A (2011). **The economic benefits of reducing physical inactivity: an Australian exampl**e. Int J Behav Nutr Phys Act.

[3] Hagger MS, Chatzisarantis NLD, Biddle SJH (2002). A meta-analytic review of the theories of reasoned action and planned behavior in physical activity: predictive validity and the contribution of additional variables. J Sport Exerc Psychol.

[4] Lonsdale C, Rosenkranz RR, Peralta LP, Bennie A, Fahey P, Lubans DR (2013). A systematic review and meta-analysis of interventions designed to increase moderate-to-vigorous physical activity in school physical education lessons. Prev Med.

[5] Standage M, Gillison FB, Ntoumanis N, Treasure DC (2013). Predicting students’ physical activity and health-related well-being: A prospective cross-domain investigation of motivation across school physical education and exercise settings. J Sport Exerc Psychol.

[6] Singapore Ministry of Education. Physical education: teaching and learning syllabus: primary, secondary and pre-university. Singapore; 2014. https://www.moe.gov.sg/docs/default-source/document/education/syllabuses/physical-sports-education/files/physical_education_syllabus_2014.pdf. Accessed 22 Sep 2017.

[7] Haynes RB, McKibbon KA, Kanani R (1996). Systematic review of randomised trials of interventions to assist patients to follow prescriptions for medications. Lancet.

[8] Stice E, Shaw H, Marti CN (2006). A meta-analytic review of obesity prevention programs for children and adolescents: the skinny on interventions that work. Psychol Bull.

[9] Cleland V, Dwyer T, Blizzard L, Venn A (2008). The provision of compulsory school physical activity: associations with physical activity, fitness and overweight in childhood and twenty years later. Int J Behav Nutr Phys Act.

[10] Brown T (2009). Systematic review of school-based interventions that focus on changing dietary intake and physical activity levels to prevent obesity: an update to the obesity guidance produced by the National Institute of health and clinical excellence. Obes Rev.

[11] Harris KC, Kuramoto LK, Schulzer M, Retallak JE (2009). Effect of school based physical activity interventions on body mass index: a meta-analysis. Am J Prev Med.

[12] Hardeman W, Johnston M, Johnston DW, Bonetti D, Wareham NJ, Kinmonth AL (2002). Application of the theory of planned behaviour in behaviour change interventions: a systematic review. Psychol Health.

[13] Miche S, Johnston M (2012). Theories and techniques of behaviour change: developing a cumulative science of behaviour change. Health Psychol Rev.

[14] McEachan RRC, Conner M, Taylor NJ, Lawton RJ (2011). Prospective prediction of health-related behaviours with the theory of planned behaviour: a meta-analysis. Health Psychol Rev.

[15] Darker CD, French DP, Eves FF, Sniehotta FF (2010). An intervention to promote walking amongst the general population based on an 'extended' theory of planned behaviour: a waiting list randomised controlled trial. Psychol Health.

[16] Ajzen I, Joyce N, Sheikh S, Cote NG (2011). Knowledge and the prediction of behavior: the role of information accuracy in the theory of planned behavior. Basic Appl Soc Psychol.

[17] Jones LW, Courneya KS, Fairey AS, Mackey JR (2005). Does the theory of planned behavior mediate the effects of an oncologist's recommendation to exercise in newly diagnosed breast cancer survivors? Results from a randomized controlled trial. Health Psychol.

[18] Sniehotta F (2009). An experimental test of the theory of planned behavior. Appl Psychol.

[19] Ajzen I (1991). The theory of planned behavior. Organ Behav Hum Decis Process.

[20] Chatzisarantis NLD, Hagger MS (2005). Effects of a brief intervention based on the theory of planned behavior on leisure-time physical activity participation. J Sport Exerc Psychol.

[21] Chatzisarantis NLD, Kamarova S, Wang J, Kawabata M, Hagger MS (2015). Developing and evaluating effects of an intervention based on the theory of planned behaviour in promoting leisure-time physical activity. Int J Sport Psychol.

[22] Faul F, Erdfelder E, Lang AG, Buchner A (2007). G*power 3: a flexible statistical power analysis program for the social, behavioral, and biomedical sciences. Behav Res Methods.

[23] Cole TJ, Belizzi MC, Flegal KM, Dietz WH (2000). Establishing a standard definition for child overweight and obesity worldwide: international survey. BMJ.

[24] Craig CL, Marshall AL, Sjostrom M, Bauman A, Booth ML, Ainsworth BE, Pratt M, Ekelund U, Yngve A, Sallis JF, Oja P (2003). International physical activity questionnaire: 12-country reliability and validity. Med Sci Sports Exerc.

[25] Norman P, Smith L (1995). The theory of planned behavior and exercise—an investigation into the role of prior behavior, behavioral intentions and attitude variability. Eur J Soc Psychol.

[26] Constructing a TPB questionnaire: Conceptual and methodological considerations**.** 2003 http://www-unix.oit.umass.edu/~aizen. Accessed 14 April 2003.

[27] Muthén, LK, Muthén B. (2017). Mplus (Version 8.0). [Computer software]. Los Angeles, CA.

[28] Drummond MF, Sculpher MJ, Torrance GW, O’brien BJ, Stoddart GL (2005). Methods for the economic evaluation of health care Programmes.

